# Experimental study on the treatment of deep miscellaneous fill foundation in Xinjiang by vibrating rod compaction method

**DOI:** 10.1038/s41598-024-66683-w

**Published:** 2024-07-12

**Authors:** Xuejun Liu, Jingchun Xue, Wei Mao, Liangfu Xie, Guangyin Du

**Affiliations:** 1 College of Civil Engineering and Architecture, Xinjiang University, Urumqi, 830017 China; 2 Xinjiang Academy of Architectural Science (Limited Liability Company), Urumqi, 83002 China; 3 Xinjiang Vocational and Technical College of Communications, Urumqi, 831401 China; 4 School of Transportation, Southeast University, Nanjing, 211189 China

**Keywords:** Vibrating rod compaction method, Fine-grained miscellaneous fill, Laboratory tests, Field tests, Vibration effects, Engineering, Civil engineering

## Abstract

Currently, the treatment of miscellaneous fill foundations, composed of a mixture of domestic garbage, construction solid waste, and natural soil, presents a significant challenge in urban peripheral engineering construction. This paper discusses the application of vibrating rod compaction technology for foundation treatment in Xinjiang. It evaluates the effectiveness of cross-section vibrating rod compaction equipment in reinforcing fine-grained miscellaneous fill foundations. The study analyzes the impact of construction disturbances caused by the insertion of the vibrating rod, monitoring horizontal stresses at various depths. Both laboratory and field tests show significant improvements: soil dry density increased by 8% to 18%, porosity decreased by 10% to 23%, compression modulus increased by 22% to 246%, and compression coefficient decreased by 8% to 70%. Additionally, cohesion (C) and angle of friction (ɸ) saw increases ranging from 7 to 38% and 3% to 25%, respectively. Below a depth of 3 m, cone tip resistance exceeded 10 MPa, and sidewall friction resistance increased to over 100 kPa, surpassing pre-treatment values. The standard penetration test results doubled stroke length compared to pre-treatment, indicating a substantial improvement in foundation bearing capacity. Surface wave tests before and after treatment showed a 15% increase in wave velocity, reflecting a more compact soil structure. The vibrating rod compaction method is innovative, energy-efficient, environmentally friendly, and economically beneficial, holding great potential for future miscellaneous fill treatments.

## Introduction

The pace of modern urban construction is accelerating rapidly; the urban population has seen a dramatic increase, and as living standards continue to rise, substantial quantities of domestic garbage and construction waste are generated^1^. In suburban areas, numerous disposal sites for household and industrial waste often merge over time with the original soil, leading to layers of mixed domestic and industrial waste and construction debris. This mixing results in a leveling process, forming what are known as miscellaneous fill foundations^1,2^. At these sites, the nature and composition of the filling materials vary due to regional activities and the original geomorphology, and the backfilling method is often characterized by random piling and disordered placement^3^. Consequently, the miscellaneous fill soil strata formed are irregular in thickness and homogeneity, with a complex composition of fillers, low strength, high compressibility, a soft texture, and very poor engineering properties. Surveys generally mandate that this soil not be used as a foundation for construction without appropriate treatment^4^. With the expansion of urban areas, the study of reinforcement methods for soil foundations containing mixed fill becomes increasingly significant. Scholars, both domestically and internationally, have extensively researched the reinforcement of miscellaneous fill soil foundations. Commonly used methods for treating these foundations include the replacement method^3^, the tamping method^4,5^, the pile foundation method^6,7^, and the grouting method^8^. Initially, the excavation and replacement method was most commonly used to strengthen the foundations; the miscellaneous soil was excavated from its original location and transported elsewhere. This treatment process not only required substantial manpower, financial, and material resources but also led to the occupation of new land resources, potentially causing new environmental pollution and necessitating secondary reinforcement^3^. The ramming method primarily utilizes the potential energy during the rammer's fall, converting it into significant kinetic energy. This energy is then transferred to the foundation soil layer through impact, causing intense vibration and pressure. Ultimately, through this repeated loading and unloading, the foundation soil is densely compacted, enhancing its bearing capacity^4^. However, this method is limited by the depth it can treat, and it generates considerable vibration and noise. The ramming process can also damage surrounding existing buildings and is only suitable for more open areas near the treatment site^5^. The pile foundation method is advantageous in engineering applications because it provides significant foundation rigidity, excellent seismic performance, low construction noise, minimal settlement, and high bearing capacity. It is particularly suitable for urban renewal projects and densely populated sites, effectively solving many of the problems encountered with miscellaneous fill in engineering. However, this method is costly, and its environmental impact can be significant due to numerous human-made factors^6^. The grouting method uses slurry to fill the pore spaces within the soil mass, but controlling the main grouting indices can be challenging due to the internal pore spaces and permeability of the miscellaneous fill. The required grouting volume is large, the reinforcement effect is hard to guarantee, and the treatment cost is high, making it unsuitable for whole-area reinforcement operations^7,8^. Therefore, proposing a new, economical, efficient, and environmentally friendly method for the treatment of miscellaneous fill foundations is of great importance.

In the 1960s, scholar Mitchell proposed the theory of vibro-consolidation for sand foundations^9^. Over the following three decades, the United States, Japan, and Europe conducted experiments on vibro compaction of foundation soils. By the 1990s, Massarsch formally introduced the vibro-replacement method, also known as the resonance compaction method^10,11^. It employs construction machinery similar to that used for domestic immersed tube filling piles. The vibrating rod, attached beneath a vibrator, is inserted into the soil through the vibrator's oscillations. By adjusting the frequency of the vibrating hammer, the vibrator, vibrating rod, and soil resonate together. This resonance causes the soil to undergo intense vibrations due to the repeated actions of sinking, pulling up, and sustained vibration^12–14^. Massarsch noted that after treatment with vibrating rod compaction, the horizontal stress within the soil mass increased substantially, and the soil's strength significantly enhanced^15^. In particular, loose saturated sand underwent complex stress conditions. Further investigations by Massarsch into the stress behavior of granular soils under cyclic loading in both plane stress and triaxial stress states revealed that friction between the soil and the vibrating rod generated horizontal compression waves, thereby augmenting the horizontal stress^16–19^. It was also found that during vibrating rod compaction, the horizontal vibration velocity exceeded the vertical velocity. Measurements of vibration at each soil layer depth showed an increase in horizontal stress after consolidation, initiating a pre-consolidation effect in the soil layers^20,21^. Since 2007, China has initiated research into vibro-compaction technology^12,13,15,24,25^, which is favored for its simple process, high construction efficiency, cost-effectiveness, and outstanding results. Over the past decade, this technology has been widely applied in the eastern coastal regions of the country for purposes such as reinforcement of reclaimed lands, mitigation of liquefaction risks in earthquake-prone areas, reduction of total and differential settlements, and improvement in the mechanical properties of liquefiable foundations^19,22–25^. In recent years, advancements in construction equipment and the maturation of vibro-compaction theory have broadened the applications of this method beyond just treating loose, liquefiable foundations. In 2019, Gao^14,26,27^and others first applied this technology to reinforce collapsible loess foundations, confirming its effectiveness in strengthening unsaturated loose soils. Subsequently, Cheng^11,28^ and others in 2021 demonstrated the innovative application of vibro-compaction technology in treating foundations with coarse-grained miscellaneous fill soils, setting a practical precedent in this field and further confirming its utility for such foundations. Compared with coarse particle mixed fill, the fine particle mixed fill is mainly composed of domestic waste, plain filled silt, and mixed with a small amount of construction waste. These soils typically exhibit poorer geological uniformity, higher compressibility and permeability, and often contain persistent pollutants. To date, there have been no reported studies or applications of vibro-compaction technology for treating fine-grained miscellaneous fill foundations.

This paper details the use of the vibrating rod compaction method in treating fine-grained miscellaneous fill foundations in the Yining area of Xinjiang. By monitoring horizontal stress alterations at different soil depths, the study analyzes the effects of construction disturbances caused by the vibrating rod on the surrounding soil mass. Laboratory tests on samples from the site and field tests were conducted to analyze changes in the soil's physical and mechanical properties after applying the vibrating rod compaction method to foundations consisting mainly of domestic garbage, construction debris, and vegetative fill powdered soil. The reinforcing effects of the method were evaluated through field tests. The outcomes of this study provide a scientific foundation and practical guidance for reinforcing fine-grained miscellaneous fill foundations using the vibrating rod compaction method.

## Conditions of test site

### Site overview

**Figure 1:**
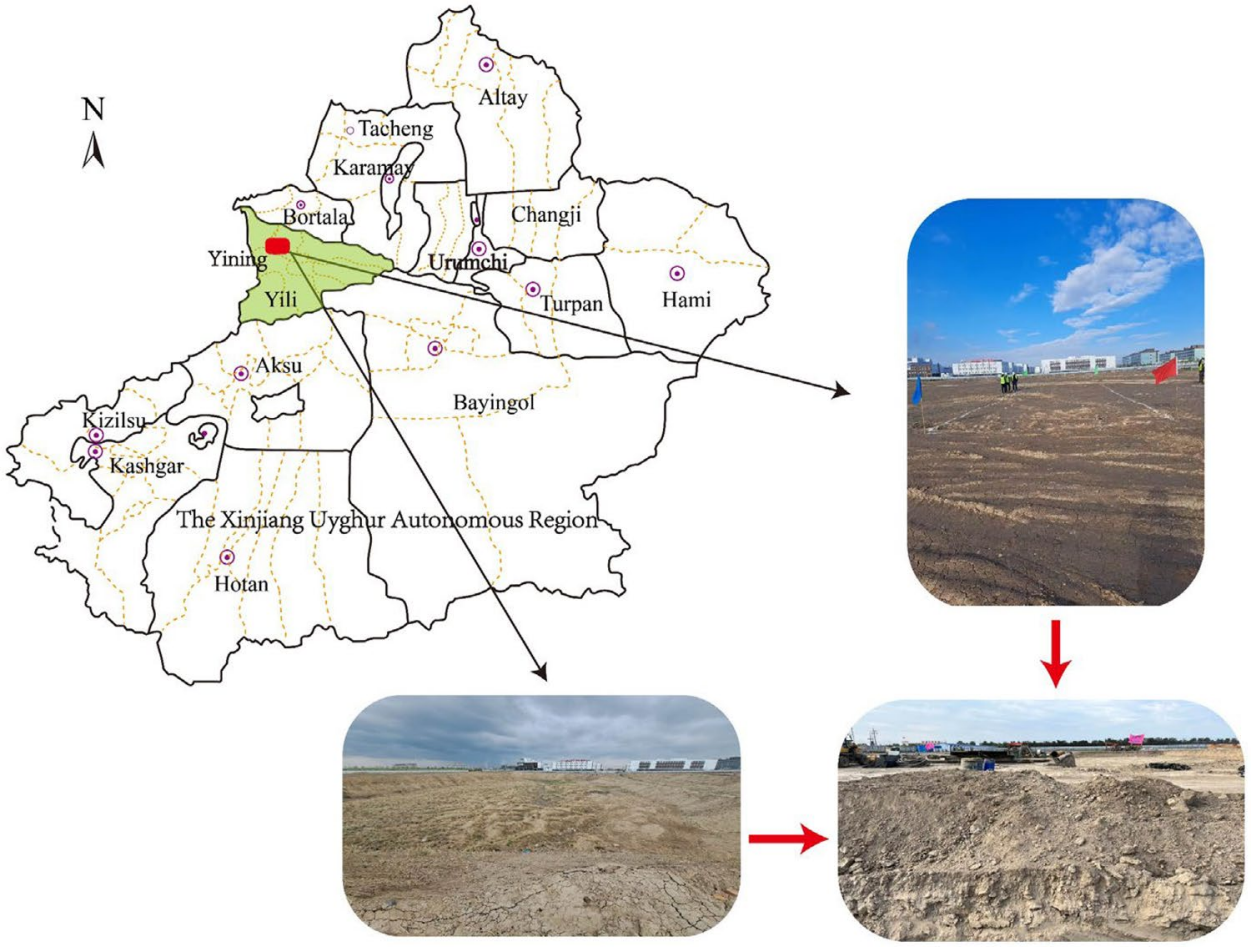
Location and topographic map of the project site in Yining, China.

The test site is located within an industrial park in Yining City, Yili Prefecture, Xinjiang. Originally, the site was vacant land featuring an alluvial ditch—the former channel of the now-dry Sule Almaty River. It spans approximately 270 m in length and varies in width from 40 to 150 m from north to south. The site's shape resembles an inverted trapezium and has been filled by the dumping of construction waste, domestic waste, and surrounding pulverized soils, creating a miscellaneous reclaimed area with a depth of nearly 10 m. The geomorphological unit at the site is singular, classified as the confluence of alluvial and floodplain terraces on the north bank of the Yili River. Its stratigraphic structure is relatively straightforward, consisting of Quaternary alluvial and floodplain deposits $\left( {Q_{4}^{al + pl} } \right)$. The groundwater level at the test site is approximately 22 m deep. Current plans propose the construction of a plant, a workers' living area, and an office building. The seismic intensity in the area is rated at VIII, with a peak ground acceleration of 0.20 g for basic seismic shaking. Figure 1 illustrates the location and geomorphology of the project site.

### Geological conditions

**Figure 2:**
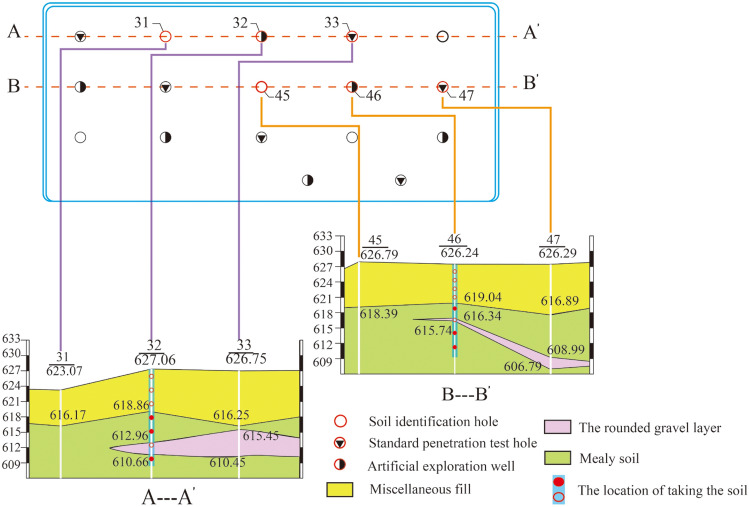
Engineering geological section of test site.

Prior to the experiment, in-situ tests and laboratory geotechnical tests were conducted in the test area. The results (Table 1) show that the site primarily consists of medium soft soil with a cover layer thickness of about 3 to 50 m. From the surface to the bottom of the borehole, the soil is divided into three layers (Fig. 2): a miscellaneous fill layer, a gravel layer, and a silt layer. The miscellaneous fill layer, composed mainly of plain fill silt, construction debris, and household waste, contains lenses of fine sand. Its thickness varies from 0.70 to 10.50 m and is discontinuously distributed, making it unsuitable for use as a natural bearing layer. The gravel layer exists in the form of lenses or interbedded with silt and gravel within the silt layer. It has an effective grain size (d_10_) average of 0.442, a limiting grain size (d_60_) average of 16.061, a curvature coefficient (C_c_) average of 0.870, and a uniformity coefficient (C_u_) average of 36.432, indicating poor grading and average soil uniformity. The thickness ranges from 0.60 to 5.00 m. The silt layer's compaction ranges from slightly dense to dense, with occasional gravel, sand lenses, or thin layers, and has relatively uniform soil quality, low dry strength, and low toughness.

**Table 1:** Physical and mechanical parameters of the soil.

Ground level	Statistical projects	Natural water content ω (%)	Natural porosity ratio e	Dry density ρ_d_ (g/cm^3^ )	Compression modulus E_s0.1–0.2_ (MPa)
Layer of miscellaneous fillings	average value	11.0	1.203	1.23	–
	(statistics) standard deviation	1.511	0.062	0.035	–
Dustbowl	average value	16.6	0.830	1.48	9.4
	(statistics) standard deviation	6.389	0.096	0.077	3.120

**Figure 3:**
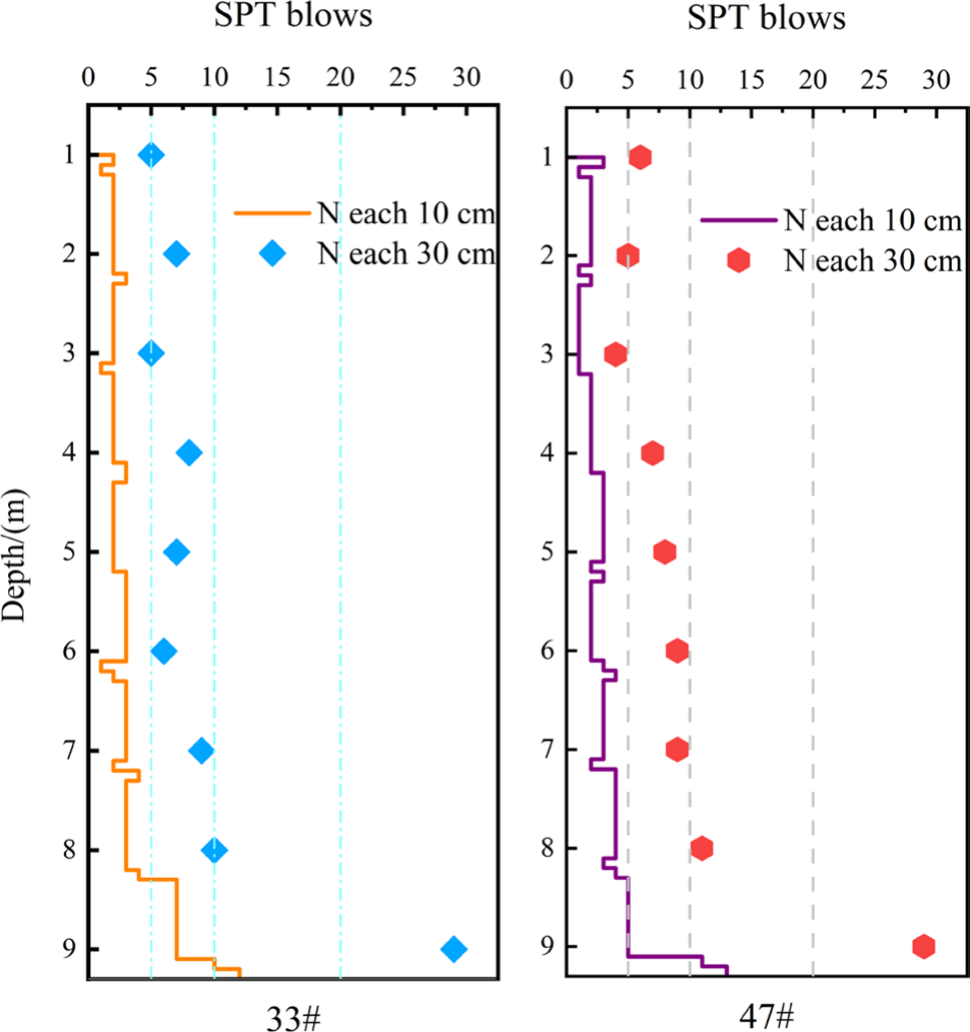
Standard penetration test data on the test site before treatment.

Data from the field standard penetration test (SPT) are depicted in Fig. 3. The test uses a 63.5 kg hammer with a drop distance of 76 cm, The number of blows is recorded for each 10 cm of penetration and adjusted according to the specifications, The cumulative number of blows penetrating 30 cm provides the standard penetration (N-value) per meter^29^.

## Test equipment and program

### Test equipment

**Figure 4:**
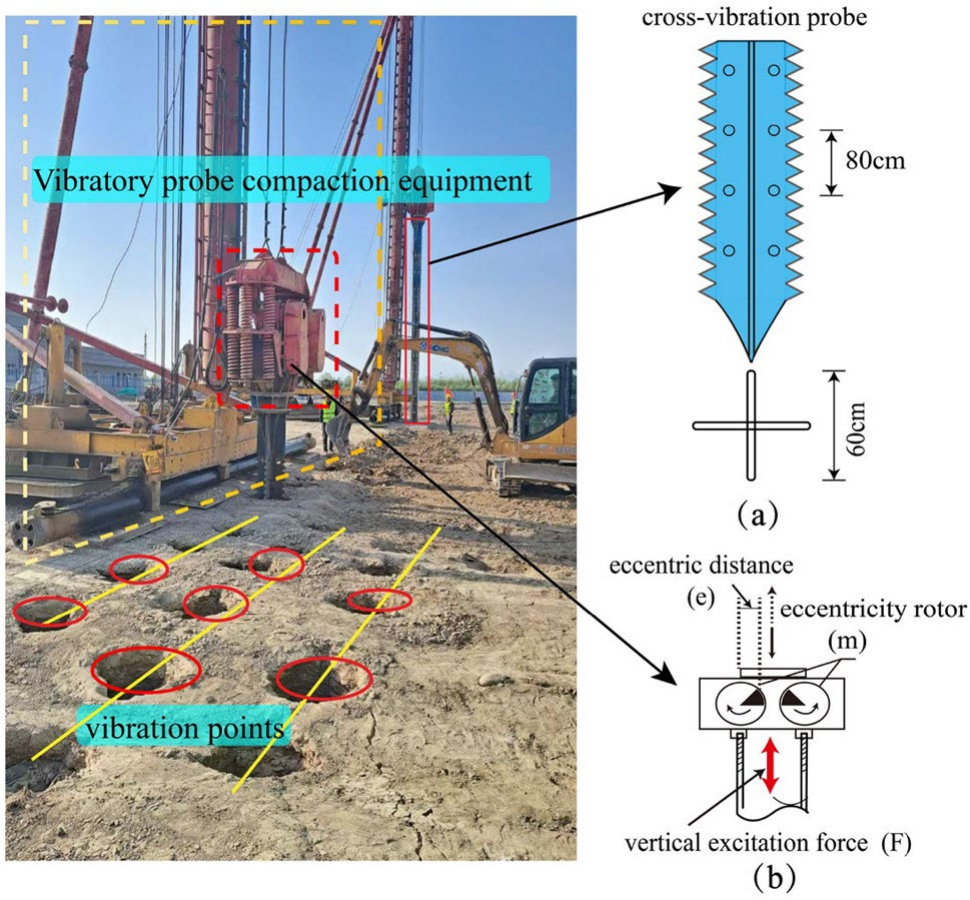
Ground treatment construction equipment (**a**) cross-vibration probe (**b**) vibration hammer.

The construction equipment used for vibrating rod compaction in the tests is depicted in Fig. 4. It primarily consists of four components: a vibrating hammer, a vibrating rod, a control system, and auxiliary equipment, the auxiliary equipment includes a traveling mechanism, a guiding frame, a generator, a pallet mechanism, among others^23–26^. The vibrating rod is constructed of two vertically intersecting steel plates with circular through-holes, cross-connected to form a cross-section shaped like a cross^14^. As illustrated in Fig. 4a, the four straight wings of the vibrating rod feature consecutive convex triangular teeth on the outer side and spiked teeth on the inner side, This design is advantageous for breaking down the soil structure and reducing the resistance during sinking^27^. The vibratory hammer construction parameters are shown in Table 2. This vibratory hammer can adjust the excitation frequency between 10 and 50 Hz, depending on the soil layer conditions, to meet the varying frequency requirements of different construction processes during the experiment. It offers high excitation force and strong penetration capability, enabling rapid sinking and withdrawal of the vibroflot to accelerate the construction progress. On-site, during the sinking and rising phases, the vibroflot's vibration frequency is usually above 30 Hz, and the penetration rate is generally controlled within 1.0 to 2.0 m per minute. When the vibroflot reaches the predetermined depth, the vibration frequency during the vibration dwell process is around 16 Hz. The hydraulic vibratory hammer, as shown in Fig. 4b, is capable of meeting the variable frequency requirements of different construction stages during the test.

**Table 2:** Vibratory hammer parameters.

Motor power (kW)	Static eccentric moment (N m)	Eccentric elock speed (r min^−1^)	Excitation force (kN)	No-load amplitude (mm)	Allowable pressure (kN)	Mass (kg)
90	460	1050	570	10.3	240	5580

Based on prior experimental research and analysis of pre-test field results, it was observed that the surface soil's compact strength decreases after vibratory treatment^27^. Consequently, an impact mill was used post-treatment to crush a large area, thereby reinforcing the surface soil and further increasing its compactness.

### Horizontal stress test of soil

**Figure 5:**
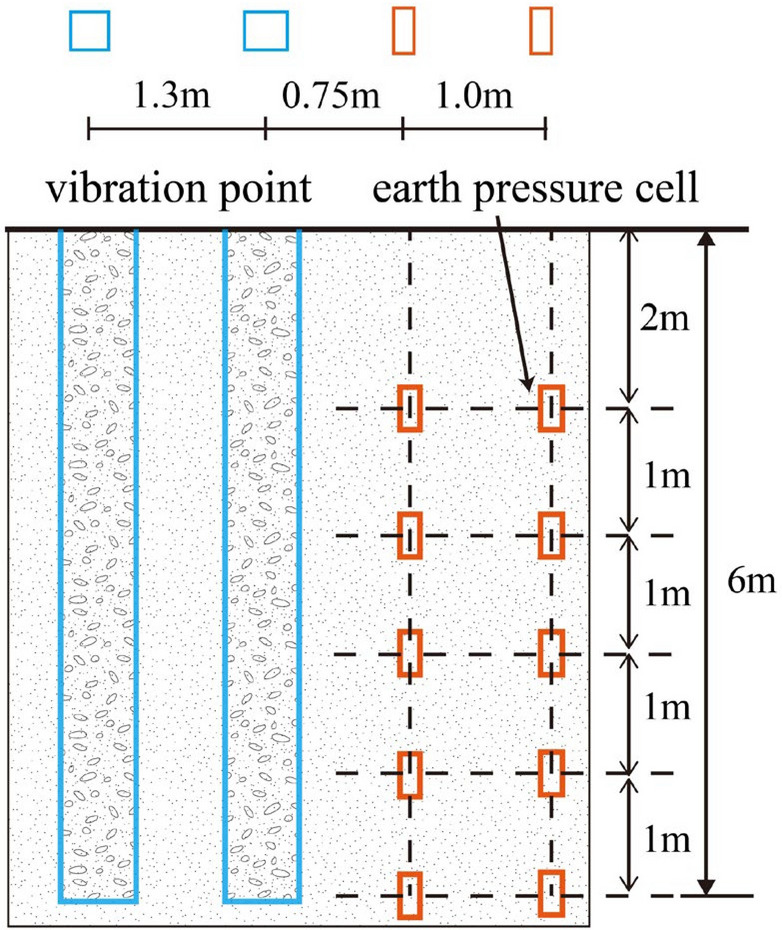
Layout of testing points in the monitoring of stress change.

**Figure 6:**
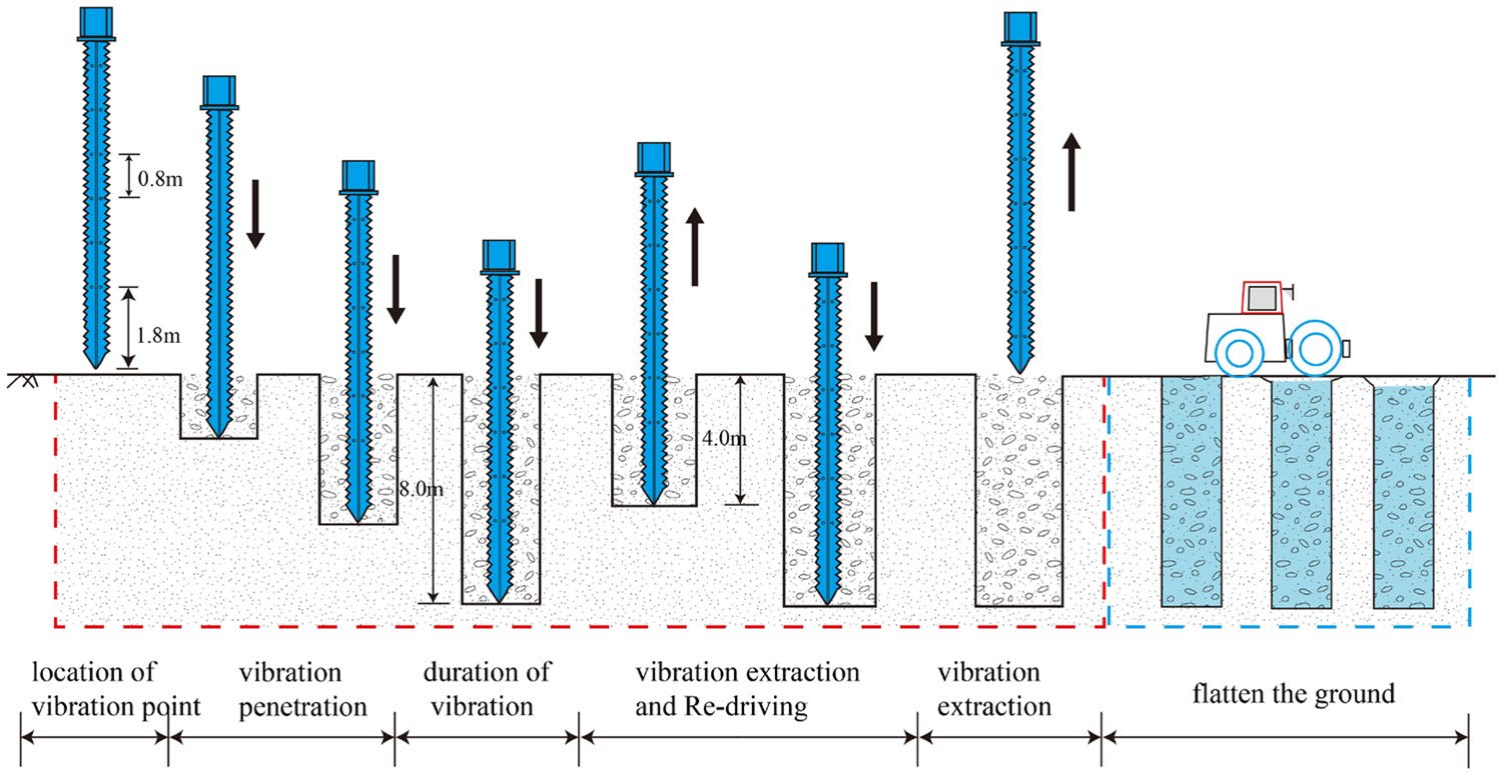
Vibratory probe compaction implementation stages in the monitoring of stress variety.

To study the horizontal stress in the soil adjacent to the vibrating rod during a single-hole construction procedure, the test area was equipped with two sets of intelligent string earth pressure boxes. These were buried to monitor changes in horizontal soil stress caused by the vibrating rod's penetration. The arrangement of the measurement points is shown in Fig. 5. The conventional construction process using the vibrating rod compaction method is documented in the literature^24–26^. The construction process for the test is divided into 8 steps, as illustrated in Fig. 6, and the lateral soil pressure change data are monitored throughout the vibration process until the test concludes.

The dynamic soil pressure sensor has a range from 0.01 MPa to 8 MPa, with an accuracy of 0.5% FS. It is circular in shape, with a radius of 3.5 cm. The data acquisition device used is the YD-6008 dynamic strain collector, which has a maximum frequency of 2200 Hz, a minimum resolution of 0.1 Hz, and an accuracy of ± 0.2 Hz. The data refresh rate can reach up to 8 channels at 100 Hz. The data collection is fast, utilizing high-speed MCU and software algorithms for real-time filtering, smoothing, anomaly marking, and data analysis. During the construction process, the vibratory hammer operates at frequencies between 10 and 50 Hz, while the maximum analysis frequency of the sensor's vibration signal is 100 Hz, which is above 50 Hz, thus meeting the requirements for vibration signal analysis.

### Laboratory soil tests

**Figure 7:**
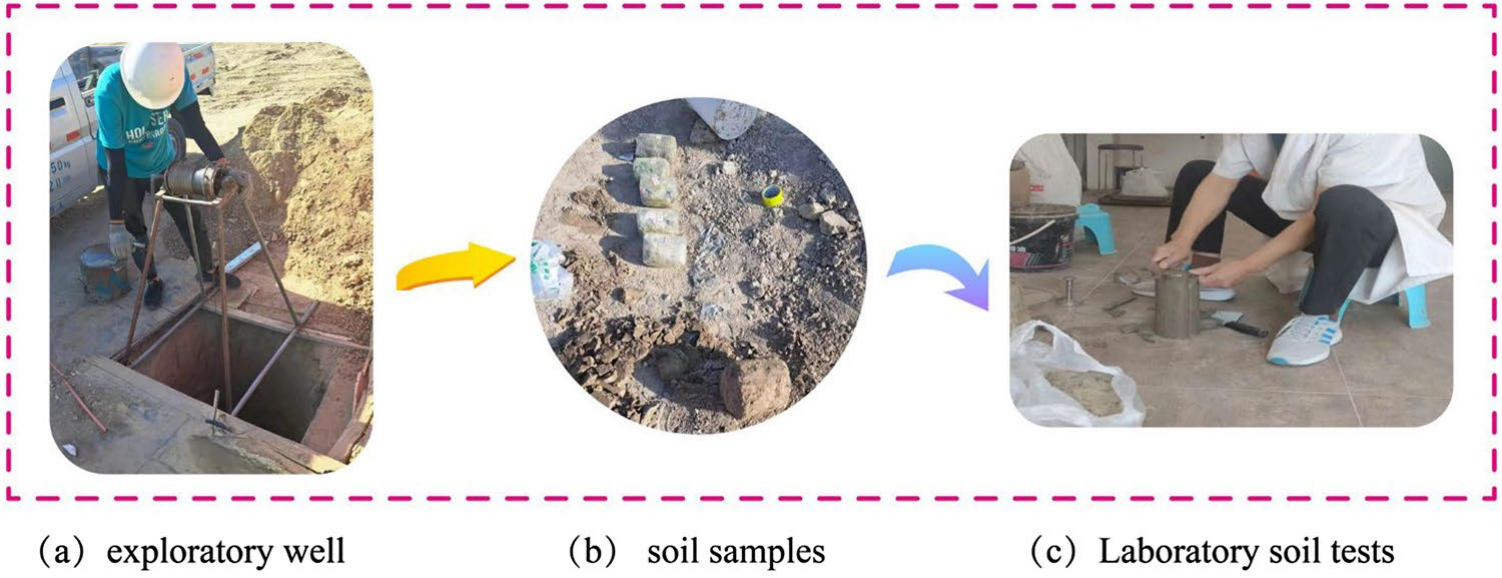
Field sampling diagram.

To investigate the physical and mechanical parameter changes in the soil before and after treatment, exploratory wells were excavated on-site. Both field and disturbed samples were manually collected at 1-m intervals (Fig. 7) and promptly transported to the laboratory for testing. Specific tests included basic parameter testing of 30-day-old laboratory soil samples to measure physical indicators such as dry density and porosity, as well as mechanical indicators like compression modulus, wetting coefficient, cohesion, and angle of internal friction.

### Tests on reinforcement effects

The test site is divided into two zones, A and B, with vibration point spacings of 1.3 m and 1.4 m, respectively, each area measuring 40 m by 10 m. The design depth for reinforcement is 8 m. After reinforcement, static cone penetration tests and standard penetration tests were conducted within the test areas.

**Figure 8:**
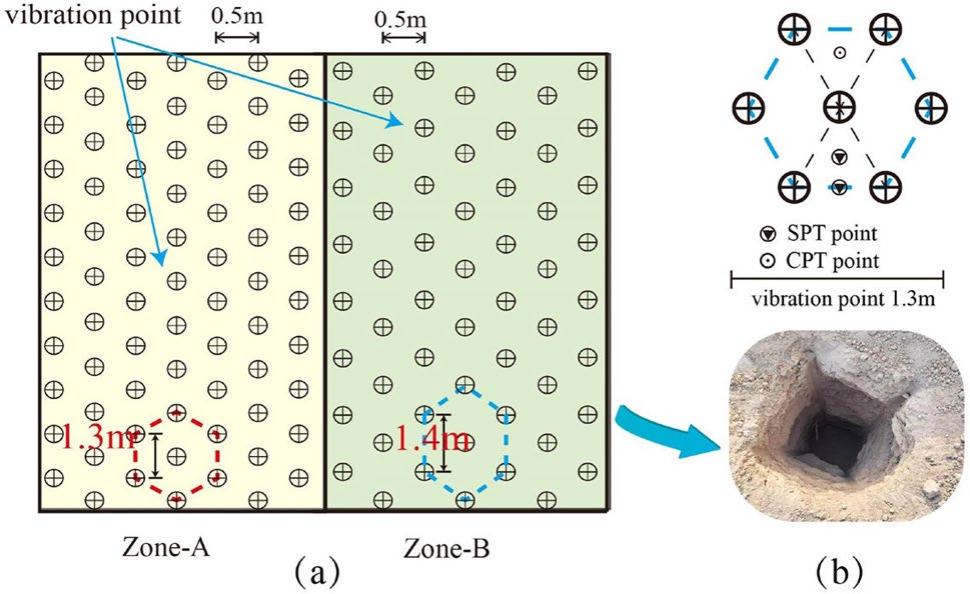
(**a**) Division of the Test Site (**b**) Layout of Dynamic Penetration Points.

Prior to and following reinforcement, transient Rayleigh surface wave tests were performed in test area A using the YL-SWS surface wave instrument. A total of 12 geophones were deployed along the measurement line at 1-m intervals, with an offset distance of 8 m. To confirm the bearing capacity of the treated miscellaneous fill layer, an immersion load test was conducted in the test area to ascertain whether the bearing capacity of the miscellaneous fill foundation meets the design criteria. The plan layout of the test area is depicted in Fig. 8.

## Analysis of test results

### Changes in soil stress

**Figure 9:**
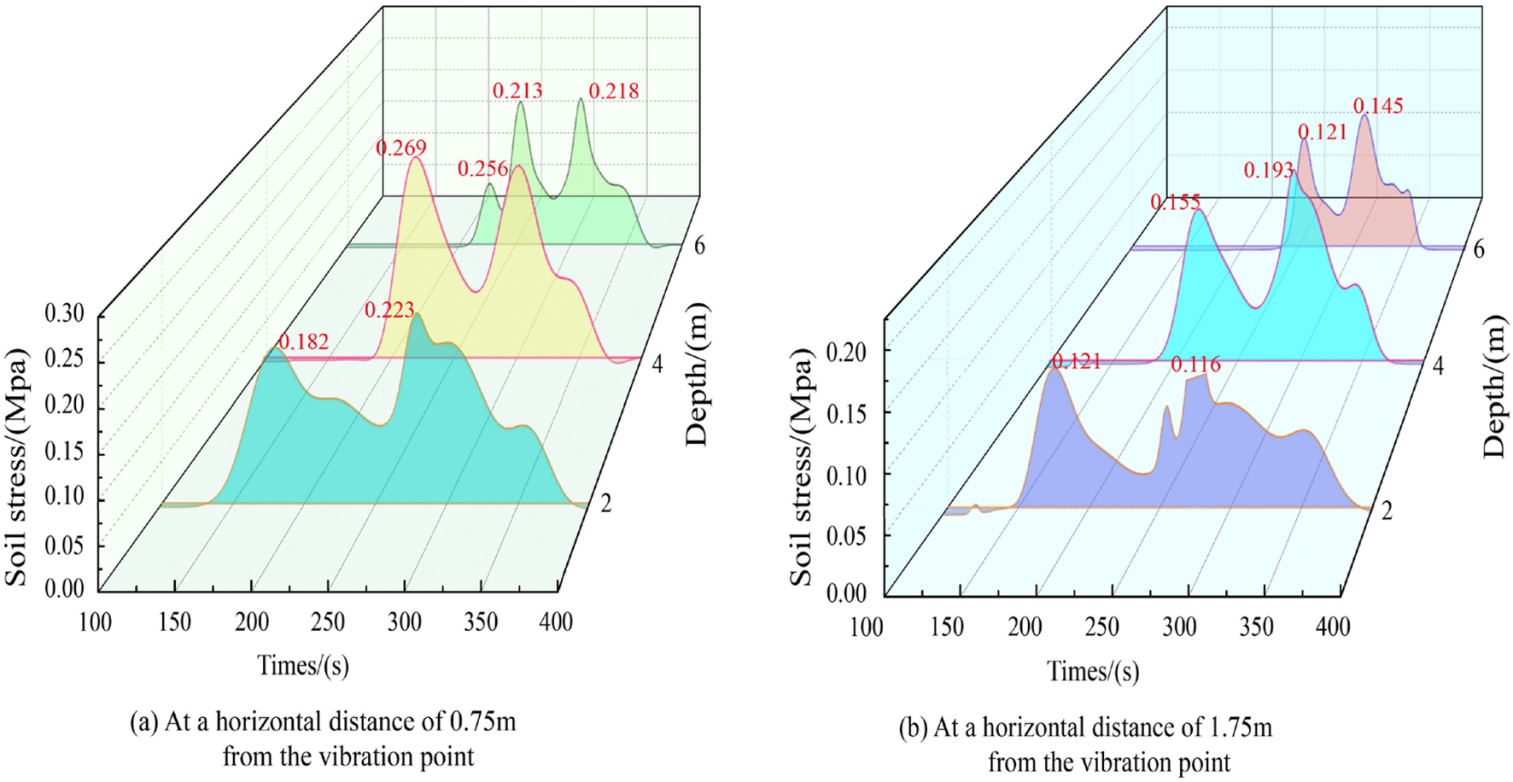
The change in horizontal stress of the soil surrounding the vibration hole during the vibrating process.

The characteristic curve illustrating the change in horizontal stress of the soil surrounding the vibration hole during the vibrating process is presented in Fig. 9. Here, positive values indicate the direction away from the vibration hole, while negative values indicate the direction toward it. For example, at a horizontal distance of 0.75 m from the vibration point, as shown in Fig. 9a, the vibrating rod induces lateral compression on the soil as it descends with high-frequency vibrations, causing horizontal displacement away from the vibration point. The sinusoidal earth pressure sensor records the change in the soil's horizontal pressure, which peaks when the vibrating rod reaches the depth of the sensor. The horizontal pressure at depths of 4 m and 6 m is greater than at 2 m, indicating that the reinforcement effect is more pronounced in deeper soil. The pressure at 4m exceeds that at 6 m, suggesting that the primary function of the vibrating rod's tip is to displace soil, thereby enabling rapid descent to the designated depth. Figure 9a,b indicate that horizontal stress slightly decreases as the length of the rod increases.

**Figure 10:**
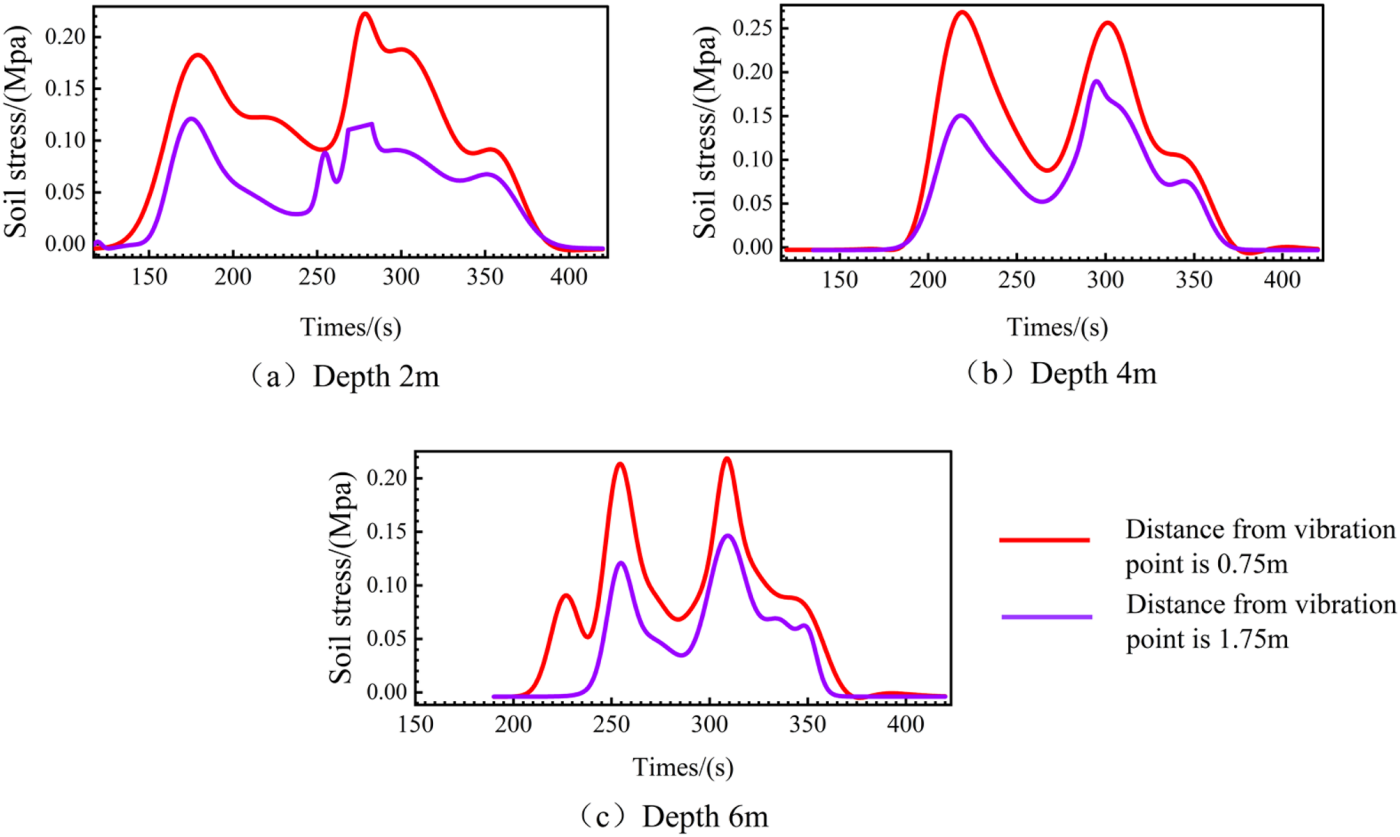
Variations in horizontal soil pressure at different distances from the vibration point at the same burial depth.

Variations in horizontal soil pressure at different distances from the vibration point at the same burial depth are depicted in Fig. 10, with subfigures (a) for a burial depth of 2 m, (b) for 4 m, and (c) for 6 m. All subfigures demonstrate that peak horizontal stress diminishes as the distance from the vibration point increases, with the horizontal stress at a distance of 1.75 m from the vibration point being less than 0.2 Mpa. The study reveals that horizontal vibration of the soil is more pronounced than vertical vibration, which results in an increase in lateral pressure and a super-consolidation effect.

Regarding the stay-vibration process, the difference in horizontal stress between the stay-vibration stage and the sinking stage is not significant when the vibrating rod reaches a depth of 4 m. This observation, along with previous field experimental studies^19^, suggests that the stay-vibration primarily serves to extend the horizontal reinforcement range.

### Changes in soil physical and mechanical indicators

**Figure 11:**
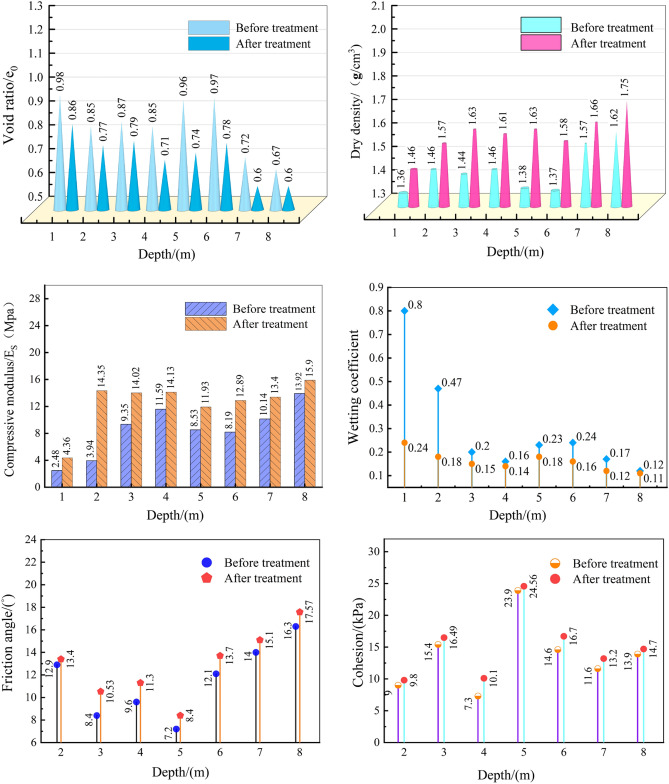
Changes of soil properties before and after treatment.

The changes in the physico-mechanical indices of the soil layer after vibrating rod compaction treatment are illustrated in Fig. 11. After vibro-compaction treatment, significant improvements are observed in the physical and mechanical indices of the soil layers at different depths. The porosity at various depths shows a notable decrease after treatment, with the maximum reduction reaching 23%, the minimum being 9%, and an average decrease of approximately 14.6%. Similarly, the dry density of soil layers at different depths is significantly enhanced, with the maximum increase being 18%, the minimum 7.3%, and an average improvement around 10%. Additionally, the compression modulus of the soil layers after treatment has also significantly increased, with the highest growth rate being 246%, the lowest 22%, and an average growth rate of 69%. The compression coefficient of soil layers at different depths has also significantly decreased, with the maximum reduction being 70% and the minimum 8%. Finally, both the cohesion (C) and the angle of internal friction (φ) of the soil layers at different depths have improved after treatment, with the maximum increase in cohesion being 38%, and the maximum increase in the angle of internal friction being about 25%. Based on the comparative analysis of the soil properties, it can be concluded that after the vibroflot compaction treatment, the physical and mechanical properties of the miscellaneous fill foundation have been significantly enhanced. The porosity of the soil layers has decreased, and their density has increased, leading to improved stability and shear strength. Overall, the uniformity of the miscellaneous fill foundation has significantly improved, and its engineering properties have been effectively enhanced.

### Evaluation of reinforcement effect

#### In situ test

**Figure 12:**
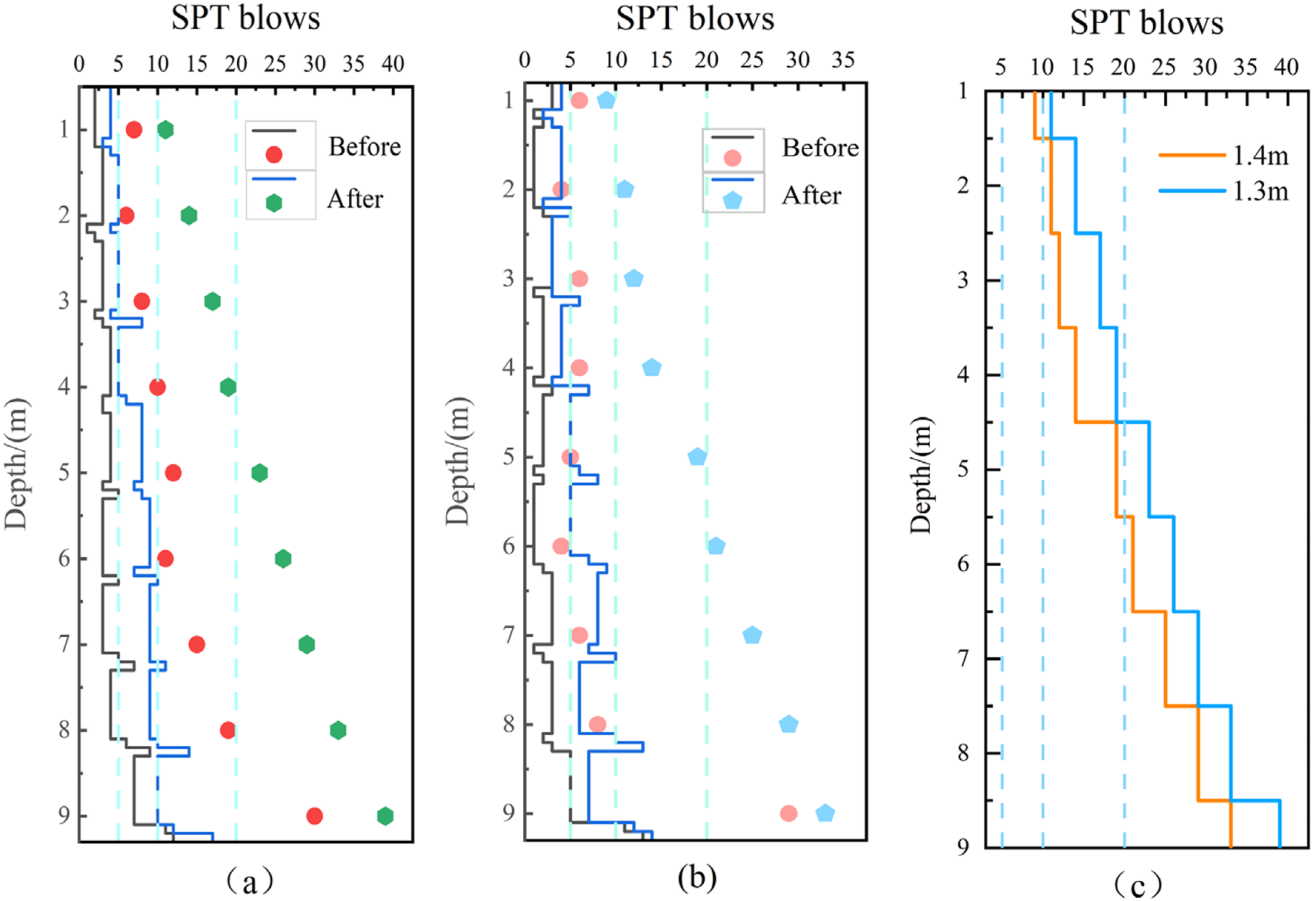
Results of Standard Penetration Test before and after Treatment (**a**) at the Test Zone with Spacing of 1.3 m (**b**) at the Test Zone with Spacing of 1.4 m (C) Contrast of different hole spacing.

The results of the standard penetration tests (SPT) conducted before and after reinforcement are presented in Fig. 12. The number of blows required per 10 cm of penetration was recorded and corrected according to the specification. The cumulative total for 30 cm of penetration is the number of blows per meter of standard penetration, denoted as N^29^. It can be observed that the number of standard penetration blows generally increases after reinforcement and further increases as the vibration point spacing decreases. Taking the vibration point spacing of 1.3 m as an example, the number of blows at the center of the triangular configuration is greater than at the center of the side length, and the number of blows at the center of the side length is greater than at the vibration point. This indicates that the compactness due to the vibrating rod in the center of the triangle and along the center of the side length benefits from the superimposed effect of the reinforcement. The strength enhancement in the subsequent 2 m is significant, with the number of standard penetration blows improving by more than double. The standard penetration increases substantially in the following 4 m, reaching a soil density classified as medium dense or higher.

**Figure 13:**
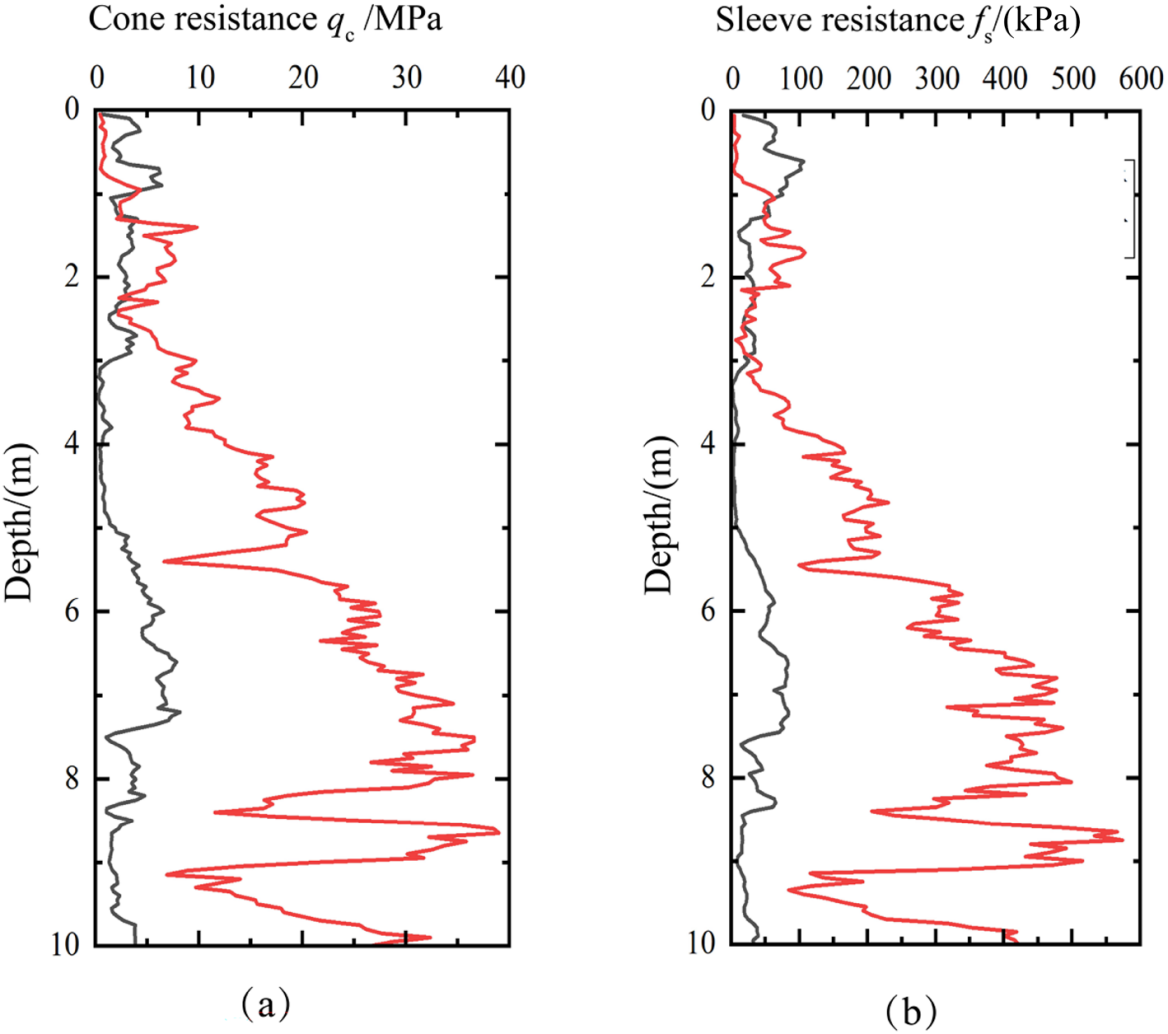
CPT data in test zone after treatment: (**a**) cone resistance and (**b**) sleeve resistance.

A dual-bridge static cone penetration test (CPT) was performed in the 1.3 m area, utilizing a 10 cm^2^ dual-bridge static probe capable of measuring both cone tip resistance (q_c_) and the sidewall resistance (f_s_) simultaneously. The test results, depicted in Fig. 13, It can be seen that after using the vibrating rod compacting method on the reinforced miscellaneous fill foundation, both the cone tip resistance and the sidewall resistance improved to varying degrees. Specifically, the cone tip resistance below 3 m is consistently above 10 MPa, and the sidewall friction resistance exceeds 150 kPa. These resistances below 3.0 m are significantly higher than those recorded before treatment, demonstrating that the vibrating rod compacting method has a notably stronger effect on deeper reinforcement.

**Figure 14:**
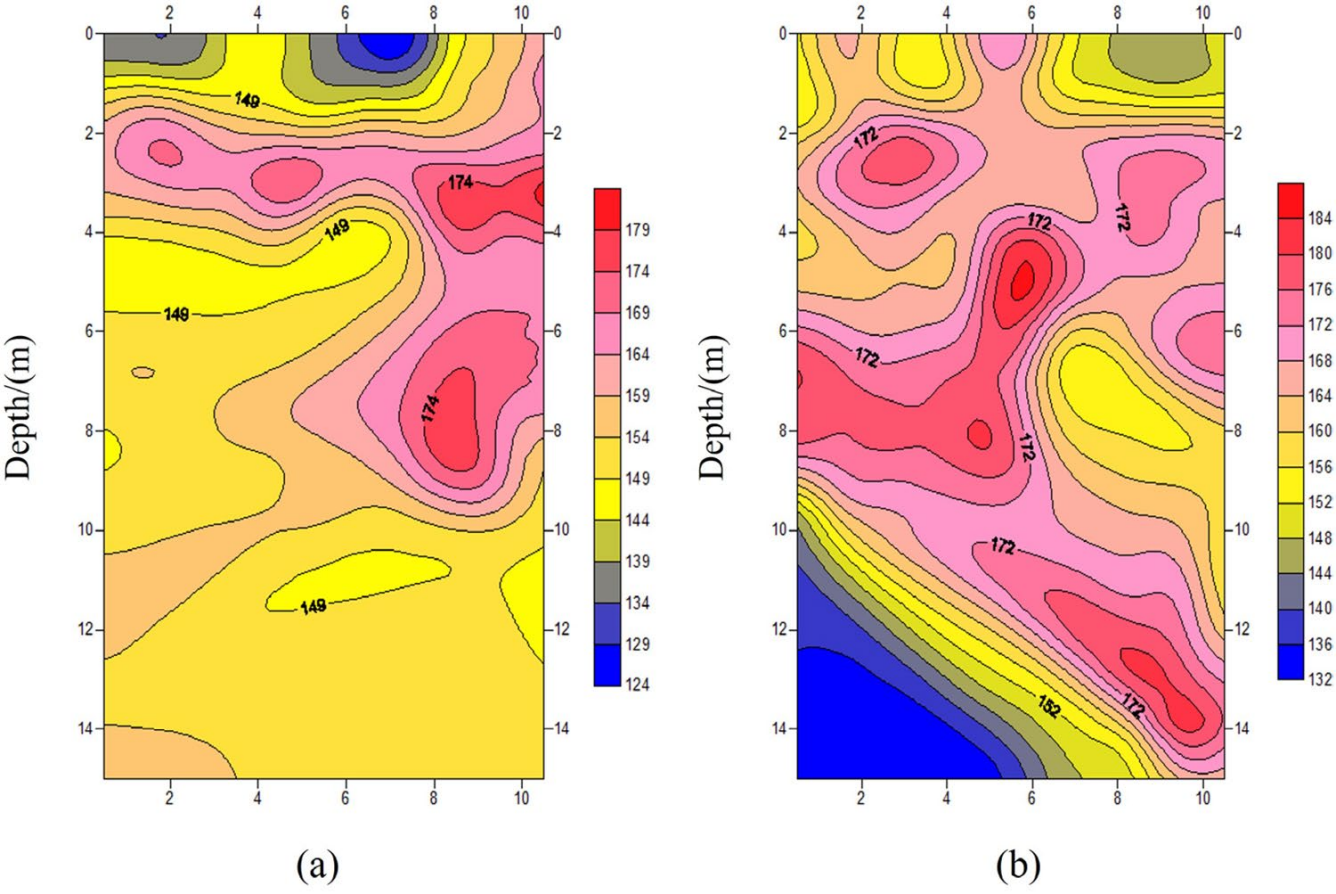
Shear wave velocity with depth at different test zones. (**a**) before treatment; (**b**) Zone 1.3 m after treatment.

The results of the ground surface wave test are shown in Fig. 14. After treatment, the wave velocity of the soil layer showed a significant improvement with increased depth. The average wave velocity before treatment was 149 m/s. Following the treatment using the vibrating rod compacting method, the wave velocity in the soil layer within the depth range of 3.0 m to 10.0 m increased significantly, with values ranging between 140 and 184 m/s, approximately 15% higher than before treatment. This indicates an improvement in the compaction of the soil layer.

#### Bearing capacity verification experiments

**Figure 15:**
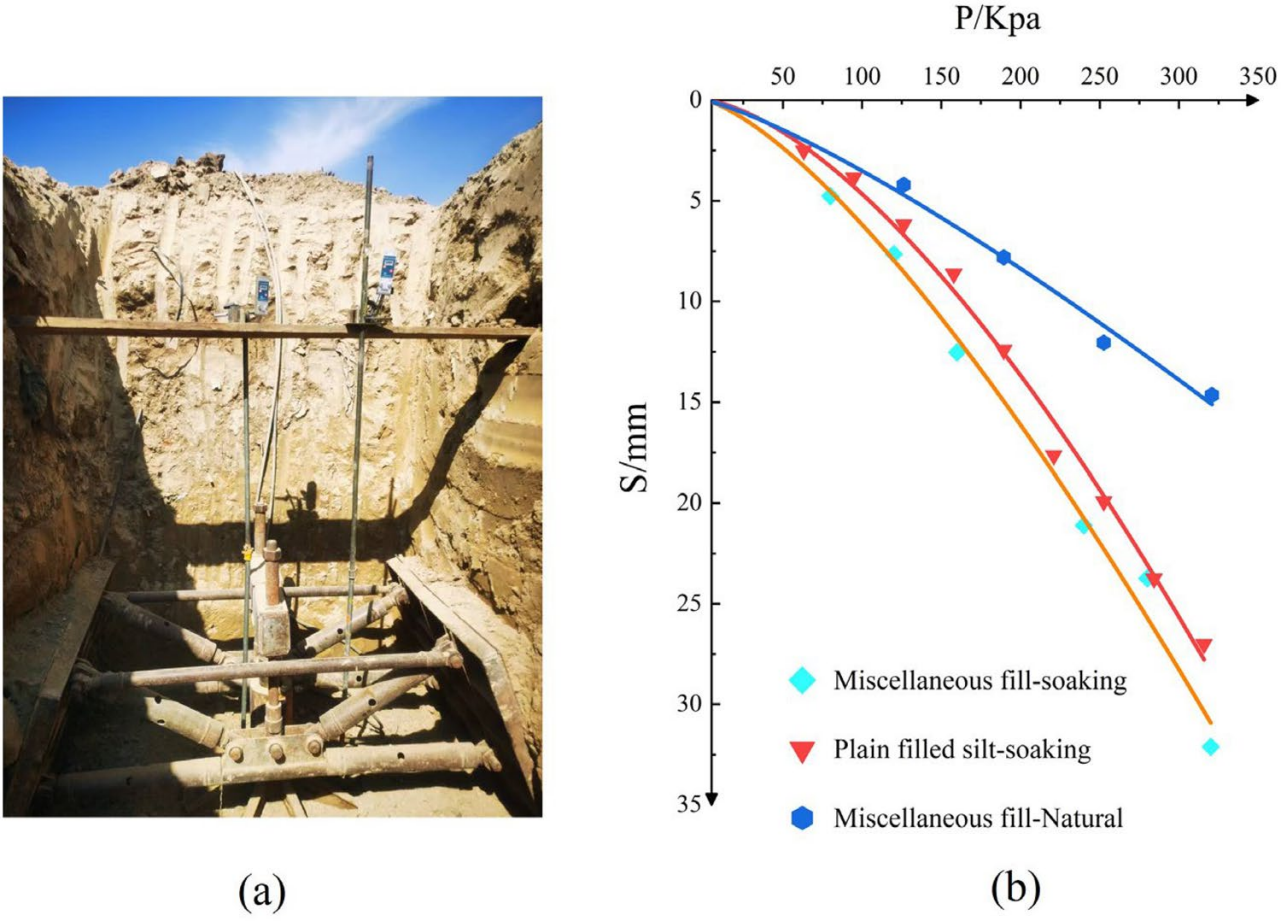
The plate load tests (**a**) experimental setup (**b**) P-S curve.

Based on the results of in-situ tests, the vibroflot compaction method showed stronger reinforcement effects at deeper layers compared to surface layers. Additionally, core samples from standard penetration tests performed at various site locations indicated diverse properties of the miscellaneous fill materials. To further verify whether the reinforced miscellaneous fill foundation meets the design requirements for bearing capacity, load tests involving shallow plate water immersion were conducted at positions with a high content of domestic waste and predominantly sandy fill. Given the loose nature, high compressibility, and permeability of the site's miscellaneous fill, two sets of water-immersed plate load tests and one set of conventional non-water-immersed plate load tests were chosen to accurately assess whether the bearing capacity of the treated miscellaneous fill foundation meets the design requirements. Figure 15a shows the plate load experimental setup, and the test depth is at 3 m underground. At the beginning of the experiment, the water immersion started at the same time, always keeping the flat plate and the surrounding times water immersion. The foundation design foundation bearing capacity is 140 kPa, according to the design requirements, detection test maximum loading force is not less than 2 times the design requirements, take 280 kPa. the load and settlement curve obtained.

The p-s curves of the three groups of load tests are shown in Fig. 15b, and their trends remain consistent. Under all levels of loading, the settlement of the bearing plate increases more uniformly with the increase of loading, and it can be stabilized in a short period of time. The load test takes the obvious lateral extrusion, bulging or continuous development of radial cracks in the soil around the bearing plate as the cutoff condition of the test^30,31^. From the p-s curve, it can be seen that when the test is cut off, the maximum displacement of the plate load test of the first group of water-soaked trash soil is 32.105 mm, and the maximum bearing capacity load is 320 kPa; the maximum displacement of the second group of water-soaked veggie soil test is 27.01 mm, and the maximum bearing capacity load reaches 315.76 kPa; the maximum displacement of the plate load test of the third group of unsoaked trash soil is 14.65 mm, and the maximum bearing capacity load reaches 315.76 kPa; the maximum displacement of the third group of unsoiled trash soil is 14.65 mm, and the maximum bearing capacity load is 315.76 kPa. The maximum displacement of the third group of unsoaked soil plate load test was 14.65 mm, and the maximum bearing capacity load was 320.76 kPa. the load test p-s curves for the three groups were gradual, with no significant bends or clear proportional limits, indicating that the foundation bearing capacity had not reached its ultimate value. According to the relevant provisions of the building foundation inspection technical standards, it can be concluded that the characteristic value of the foundation bearing capacity in the three tests reached at least 140 kPa, satisfying the design requirements. The analysis of the three load tests demonstrates that the vibroflot compaction method effectively reinforces the miscellaneous fill foundation, significantly enhancing its bearing capacity.

## Conclusion

This paper details the use of the vibrating rod compaction method in the treatment of fine-grained miscellaneous fill foundations in the Yining area of Xinjiang. The reinforcing effects of the method were evaluated through field tests. The results can be summarized as follows:

1. The use of vibrating rod compaction technology for the reinforcement of miscellaneous fill foundations—comprising vegetative soil, powdered soil, domestic garbage, and construction debris, mostly fine particles—has proven effective. As the vibrating rod sinks into the ground with high-frequency vibrations, the soil surrounding the vibrating point is subjected to lateral extrusion and vertical shear forces. This action results in soil displacement away from the vibrating point. Data from buried soil pressure sensors indicate a clear lateral extrusion effect on the soil around the vibrating point. Soil horizontal stress decreases as the distance from the vibrating point increases and rises with the depth of the vibrating rod's penetration.
2. Laboratory soil property experiments have shown that the vibrating rod compaction method significantly improves the physical and mechanical indices of fine-grained miscellaneous fill treated soil layers. Post-treatment improvements include a reduction in pore ratio by 10% to 23%, an increase in dry density by 8% to 18%, a substantial rise in compression modulus by 22% to 246%, and a reduction in compression coefficient by 8% to 70%. The cohesion (C) and internal friction angle (φ) increased by approximately 7% to 38% and 3% to 25%, respectively. When used in conjunction with impact milling for joint construction to reinforce foundations, significant enhancements in soil density were observed for both surface and deeper layers.
3. Arranging vibration holes in a hexagonal pattern resulted in a significant increase in the number of standard penetration test hits compared to the soil layer before treatment, whether at the hexagon's side center or at the three-point shape center. Both cone tip resistance and sidewall resistance from static contact tests showed notable improvement after treatment. Shear wave velocity and surface wave measurements before and after treatment indicated that post-treatment soil layer wave velocities increased with depth, signifying improved compactness.
4. Following reinforcement with the vibrating rod compaction method, the pore ratio of the soil layer is reduced, and enhancements in both Rayleigh wave velocity and foundation bearing capacity are observed. According to the design requirements, this method renders the treated soil suitable as a natural foundation for roads, fields, and pipelines. For buildings (structures) with few stories, uniform load distribution, and moderate requirements for bearing capacity and deformation, shallow foundations can be directly employed, serving as a foundation holding layer. Compared to foundation treatment methods such as dynamic compaction and reclamation, vibrating rod compaction stands out as an economical, green, low-carbon, and environmentally friendly foundation treatment technique.

## Data Availability

All data generated or analysed during this study are included in this published article [and its supplementary information files].

## References

[1] Jialei W, Jinbao H, Xinyan M (2021). Field test research on strengthening deep and thick backfilled foundation soil by 12000 kN m high energy dynamic compaction. Chin. J. Undergr. Space Eng..

[2] Zhang F, Zhang L, Zhou T (2020). An experimental study on settlement due to the mutual embedding of miscellaneous fill and soft soil. Adv. Civ. Eng..

[3] Bin F, Hai Tao B, Ling Quan C (2022). Case study of ground treatment for thick miscellaneous fill site. J. Ground Improv..

[4] Hao LI, DongJia L, ChaoQun H (2012). A case study of the effect of dynamic compaction on miscellaneous fill foundation treatment. J. Hefei Univ. Technol. (Nat. Sci.).

[5] Xiang QL (2003). Case history for treatment of miscellaneous fill by dynamic compaction. Geotech. Investig. Survey..

[6] Pengfei F (2009). Comparison text studies on the load bearing characteristics of post-grouted bored piles in soft soil. J. Eng. Geol..

[7] Deliang Z, Wenjun W, Xinyu X (2020). Reinforcement case of high-pressure rotary jet grouting pile for thick construction waste miscellaneous fill foundation. Build. Struct..

[8] Yan W, Wei X, Qingchun F (2016). Ground grouting reinforcement techniques and application in a Beijing metro project. Mod. Tunnel. Technol..

[9] Mitchell JK (1970). In-place treatment of foundation soils. J. Soil Mech. Found. Div..

[10] Massarsch KR (1991). Deep soil compaction using vibratory probes. Astm Spec. Tech. Publ..

[11] Yuan C, Ye Y, Gang S (2023). Field tests on the improvement effect of miscellaneous fills using the vibratory probe compaction method. China J. Highw. Lransp..

[12] Yuan C, Songyu L, Guangyin D (2015). Application and recent research progress of vibratory probe compaction method. China Earthq. Eng. J..

[13] Changhui G, Guangyin D, Songyu L (2022). Study on assessment method and application scope of vibratory probe compaction method using CPTU-based soil classification method. China Civ. Eng. J..

[14] Changhui G, Guangyin D, Songyu L (2022). Influence of deep vibratory compaction on the horizontal stress change of collapsible loess. Rock Soil Mech..

[15] Yuan C, Songyu L, Hehua Z (2016). Experiment of construction disturbance and factors influencing vibratory probe compaction effect on liquefaction site treatment. China J. Highw. Transp..

[16] Massarsch KR, Fellenius BH (2002). Vibratory compaction of coarse-grained soils. Can. Geotech. J..

[17] Massarsch KR, Wersäll C, Fellenius BH (2020). Discussion: Horizontal stress increase induced by deep vibratory compaction. Proc. Inst. Civ. Eng.-Geotech. Eng..

[18] Massarsch KR, Wersll C, Fellenius BH (2019). Horizontal stress increase induced by deep vibratory compaction. Geotechn. Eng..

[19] Yuan C, Yupeng F, Xinjun G (2021). Reinforcement range of vibratory probe compaction for liquefaction site treatment based on the principle of energy dissipation. China J. Highw. Transp..

[20] Massarsch KR, Fellenius BH (2019). Evaluation of vibratory compaction by in situ tests. J. Geotechn. Geoenviron. Eng..

[21] Massarsch KR, Wersäll C (2020). Vibratory plate resonance compaction. Proc. Inst. Civ. Eng.-Geotech. Eng..

[22] Qian G, Guangyin D, Changhui G (2019). Experimental study on influence of clay lens on compaction effect of resonant compaction method. J. Southeast Univ..

[23] Yuan C, Jie H, He-hua Z (2019). Vibratory probe compaction effect on silty foundation treatment. China J. Highw. Transp..

[24] Yuan C, Song-yu L (2013). Application of resonant vibro-compaction in treatment of liquefiable sites. Chin. J. Geotechn. Eng..

[25] Song-Yu L, Yuan C (2012). Resonance compaction method for highway ground improvement at liquefaction site. China J. Highw. Transp..

[26] Song-yu L, Guang-yin D, Zhong-liang M (2020). Field tests on improvement of collapsible loess by vibratory probe compaction method. Chin. J. Geotech. Eng..

[27] Biao Z, Ding-wen Z, Song-yu L (2021). Experimental study on construction parameters of collapsible loess foundation treated by vibrating rod compaction method. Chin. J. Geotech. Eng..

[28] Zhuang Z, Du G, Liu S (2023). Field study on the densification of construction and demolition waste fill using vibratory probe compaction technique. Environ. Earth Sci..

[29] GB National Standard (2019). Standard for Geotechnical Testing method: GB/T 50123–2019.

[30] Yong-hui L, Wei-dong W, Shi-jin F (2022). Field study on treatment of collapsible silt for high-fill airport project. Chin. J. Geotechn. Eng..

[31] Code for Design of Building Foundation (2011). GB 50007–2011.

